# Cyanuric acid hydrolase: evolutionary innovation by structural concatenation

**DOI:** 10.1111/mmi.12249

**Published:** 2013-05-20

**Authors:** Thomas S Peat, Sahil Balotra, Matthew Wilding, Nigel G French, Lyndall J Briggs, Santosh Panjikar, Nathan Cowieson, Janet Newman, Colin Scott

**Affiliations:** 1 CSIRO Materials, Science and EngineeringParkville, Vic., 3801, Australia; 2 CSIRO Ecosystem SciencesBlack Mountain, Canberra, ACT, 2601, Australia; 3 Australian SynchrotronClayton, Vic., 3168, Australia; 4 Department of Biochemistry and Molecular Biology, Monash UniversityVic., 3800, Australia

## Abstract

The cyanuric acid hydrolase, AtzD, is the founding member of a newly identified family of ring-opening amidases. We report the first X-ray structure for this family, which is a novel fold (termed the ‘Toblerone’ fold) that likely evolved via the concatenation of monomers of the trimeric YjgF superfamily and the acquisition of a metal binding site. Structures of AtzD with bound substrate (cyanuric acid) and inhibitors (phosphate, barbituric acid and melamine), along with mutagenesis studies, allowed the identification of the active site. The AtzD monomer, active site and substrate all possess threefold rotational symmetry, to the extent that the active site possesses three potential Ser–Lys catalytic dyads. A single catalytic dyad (Ser85–Lys42) is hypothesized, based on biochemical evidence and crystallographic data. A plausible catalytic mechanism based on these observations is also presented. A comparison with a homology model of the related barbiturase, Bar, was used to infer the active-site residues responsible for substrate specificity, and the phylogeny of the 68 AtzD-like enzymes in the database were analysed in light of this structure–function relationship.

## Introduction

The deployment of the *s-*triazines, such as atrazine (1-chloro-3-ethylamino-5-isopropylamino-2,4,6-triazine), into the environment has resulted in the evolution of new atrazine catabolic pathways in bacteria (Wackett, 2009). These pathways, and the enzymes that comprise them, have garnered considerable attention in light of their potential application in the bioremediation of atrazine (Udikovic-Kolic *et al*., 2012), which is a potential human carcinogen, endocrine disrupter and teratogen (Wiegand *et al*., 2001; MacLennan *et al*., 2002; Hayes *et al*., 2003). *s*-Triazine degrading systems are also model systems for the study of evolving enzymes and pathways (Wackett, 2009; Noor *et al*., 2012; Udikovic-Kolic *et al*., 2012).

**Fig. 1:**
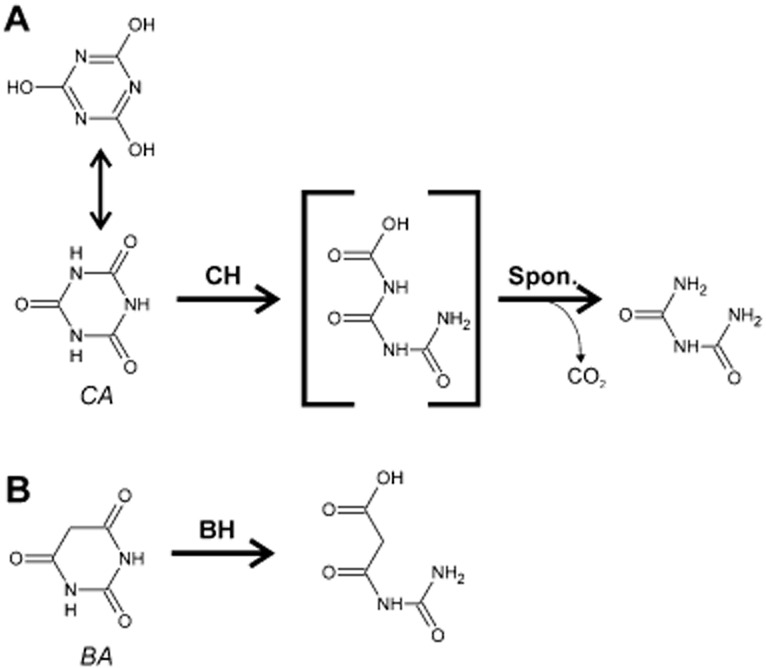
Enzymatic hydrolysis of cyanuric acid and barbituric acid. A. Cyanuric acid (*CA*) tautomerizes between its trihydro- and triketo-forms, the latter acting as the substrate for cyanuric acid hydrolase (CH). The product of hydrolysis is 1-carboxybiuret, which spontaneously decomposes to biuret (liberating CO_2_). B. Barbituric acid (*BA*) is hydrolysed by barbiturate hydrolase (BH) to form 3-oxo-3-uridopropionate.

Atrazine is mineralized in bacteria by a series of hydrolases, with the first three hydrolytic steps resulting in the dechlorination and deamination of atrazine to produce cyanuric acid (CA; 2,4,6-trihydroxy-1,3,5 triazine; Seffernick *et al*., 2012), which is then hydrolysed by the ring-opening amide hydrolase, cyanuric acid hydrolase (AtzD; E.C. 3.5.2.15). AtzD catalyses the hydrolytic ring opening of CA to yield 1-carboxybiuret which then spontaneously decomposes to biuret and carbon dioxide (Fig. 1A; Seffernick *et al*., 2012). Although CA is a naturally occurring compound, occasionally formed during oxidative damage of DNA (Wackett, 2009), its environmental abundance has increased markedly since the introduction of synthetic *s*-triazine compounds.

AtzD, is the archetype for a family of ring-opening amidases (Seffernick *et al*., 2012) that includes a related CA hydrolase (TrzD; 56% identical to AtzD; Karns, 1999) and barbiturase (Bar; E.C. 3.5.2.1; Soong *et al*., 2002; Seffernick *et al*., 2012). Barbituric acid (2,4,6-trihydroxy-1,3-pyrimidine; BA), is an intermediate in the oxidative degradation of pyrimidines (Soong *et al*., 2002). Pyrimidines are degraded reductively in eukaryotes and most prokaryotes (Soong *et al*., 2001); however, an oxidative pathway was described from a small number of eubacteria in the 1950s in which pyrimidines are first oxidized to BA, which is then hydrolysed by Bar and ureidomalonase to yield malonic acid and urea (Soong *et al*., 2001). This metabolic pathway is rare, as AtzD/Bar homologues were described in only 3% of 6423 surveyed genomes in a recent study (Seffernick *et al*., 2012).

Bar catalyses an equivalent ring-opening reaction to that of AtzD, using BA as substrate and yielding the stable product 3-oxo-3-ureidopropanoate (Soong *et al*., 2002; Fig. 1B). BA differs from CA at just a single position in the ring (*C*5 in BA and *N*5 in CA; Fig. 1A and B). It is therefore perhaps surprising that BA is an inhibitor, rather than a substrate, for AtzD (Seffernick *et al*., 2012) and that CA is an inhibitor of Bar (Soong *et al*., 2002). With no available structure for AtzD or Bar, the determinants of specificity were not identified.

Early studies of this family suggested that these enzymes were members of the metal-dependent amidohydrolase family; Soong and co-workers were able to inactivate Bar by treatment with a chelator and the activity was restored upon incubation with Zn^2+^ (Soong *et al*., 2002). However, more recent work from Seffernick *et al*. (2012) demonstrated that this family is unrelated to amidohydrolases, forming a discrete evolutionary class of their own. Moreover, it was suggested that the newly defined family was metal-independent.

Herein, we describe the first X-ray structure for this family, an unusual concatenated knot fold (which we term the ‘Toblerone’ fold), that possesses a threefold rotational symmetry that extends to the active-site architecture. Structures of AtzD with the substrate and inhibitors, and kinetic and mutagenesis analysis of these with wild-type and mutant proteins are presented. We identify Lys42 and Ser85 as a plausible catalytic dyad and hypothesize a catalytic mechanism consistent with that assignation.

## Results

### Structure of AtzD

**Fig. 2:**
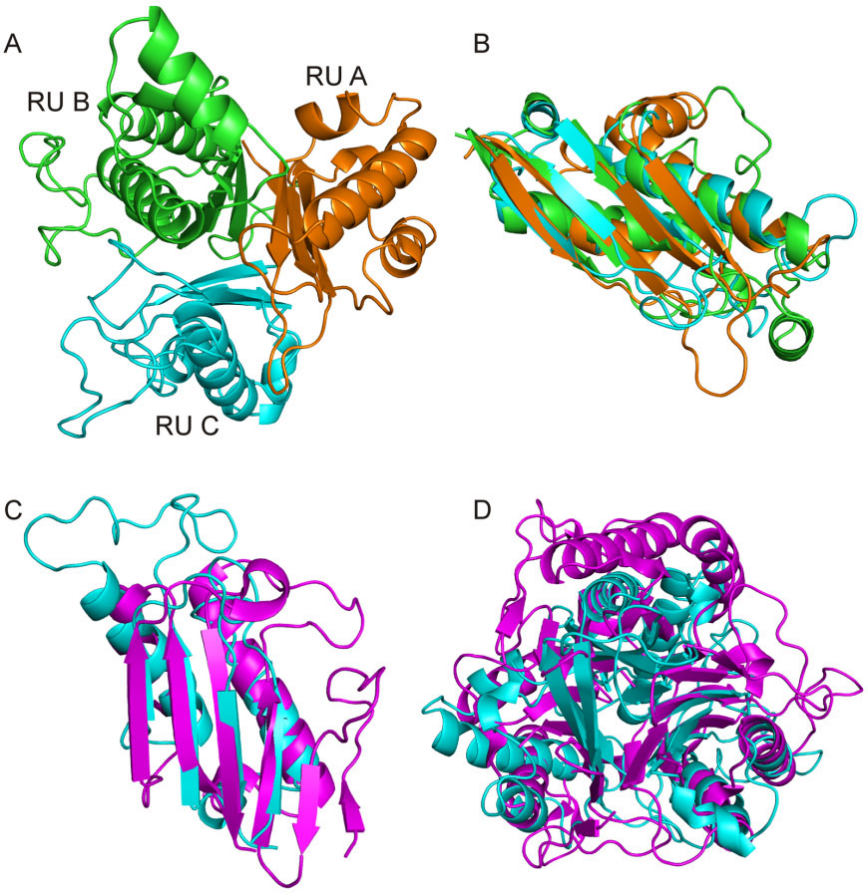
Structure of AtzD monomer and comparison to the perchloric acid soluble protein (PSP). A. Structure and substructure of an AtzD monomer. The RUs are coloured as follows: RU A (residues 2–104) is orange, RU B (residues 113–250) is green and RU C (residues 256–364) is cyan. B. Overlay between RUs A, B and C (coloured as Fig. 2A). C. PSP (PDB: 3K0T) monomer (magenta) superposed with RU C of AtzD (cyan). D. A trimer of PSP (magenta) superposed with a monomer of AtzD (cyan).

The AtzD monomer is a unique arrangement of a core of 12 β-strands with six helices on the outside (Fig. 2A). This structure is formed from three ‘repeating units’ (RUs) that share the same structure as one another; they have been designated RU A (residues 3–104), B (residues 113–250) and C (residues 256–364). Each RU is comprised of a four strand antiparallel β-sheet and two helices. This arrangement of RUs confers threefold rotational symmetry in the AtzD monomer reminiscent of a ‘Toblerone’ Bar (viewed end on; Fig. 2A), and it is proposed that this new fold be termed the Toblerone fold. The three RUs of the monomer align with the following root mean square deviation (rmsd) over the Cα atoms and sequence identities: B to A = 3.0 Å/9.3%, C to A = 2.3 Å/16.2%, C to B = 2.2 Å/17.8% (Fig. 2B).

There were no homologues to the full-length AtzD in the PDBe (using SSM). However, a single RU has homology with proteins of the YgjF superfamily: perchloric acid-soluble protein (PSP) from *Pseudomonas syringae* (PDB: 3K0T), YGJF from *Streptococcus pyogenes* (PDB: 3EWC) and RutC from *Escherichia coli* (PDB: 3V4D). PSP overlays an AtzD RU with a rmsd of ∼ 1.3 Å over 109 residues (sequence identity ∼ 16.5%; Fig. 2C), notwithstanding that the YgjF proteins have a six strand antiparallel β-sheet (rather than four). YgjF proteins form homo-trimers that align poorly with the structure of an AtzD monomer (Fig. 2D), due to the two additional β-strands per monomer/RU.

**Fig. 3:**
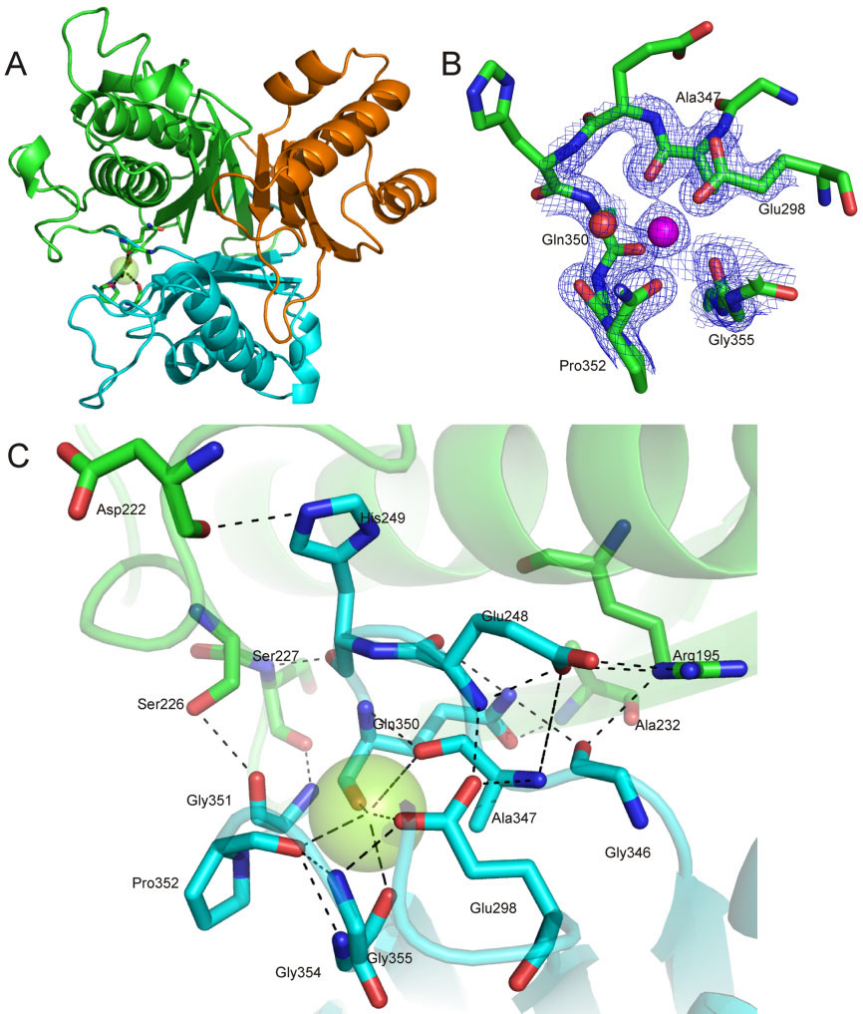
The metal binding site of AtzD. A. The position of the bound metal (green sphere) in the AtzD monomer (coloured as per Fig. 2A). B. The bound metal (magenta sphere), the metal-binding residues, the water ligand (red sphere) and their electron densities are shown. C. The interactions between the metal, metal-stabilized loop and the surrounding protein.

AtzD possesses a single metal binding site per monomer, in RU C, for which there is no equivalent in the YgjF family of proteins (Fig. 3A–C; Zhang *et al*., 2010; Knapik *et al*., 2012), but which is completely conserved in 90% of the AtzD homologues currently available (data not shown). The metal binding site consists of backbone carbonyls from residues Ala347, Gln350, Pro352, Gly355, the δ-carboxylate of Glu298 and a water molecule. The octahedral co-ordination, electron density and B factor associated with the cation suggest that the metal bound by AtzD is likely to be magnesium (Dudev *et al*., 1999; Fig. 3D). Anomalous scattering data show that the native metal ion is either magnesium or sodium (Supplemental Fig. S1). Unfortunately, it is not possible to distinguish between Na^+^ and Mg^2+^ by anomalous scattering as both cations have an identical number of electrons and the theoretical anomalous difference at accessible X-ray wavelengths is consequently near identical. However, it is certain from these data that the density found is not Zn^2+^, nor is it any other transition metal. The metal appears to stabilize an extended loop that contains residues that make polar contact with amino acids in the adjacent RU (Fig. 3B and C), which may stabilize the β-strand that holds Lys296 in the active site and contribute to the stability of the enzyme more generally. In addition, this highly ordered structure orients Glu348 in an interaction with Arg195, which sits in the active site (Fig. 3C).

**Fig. 4:**
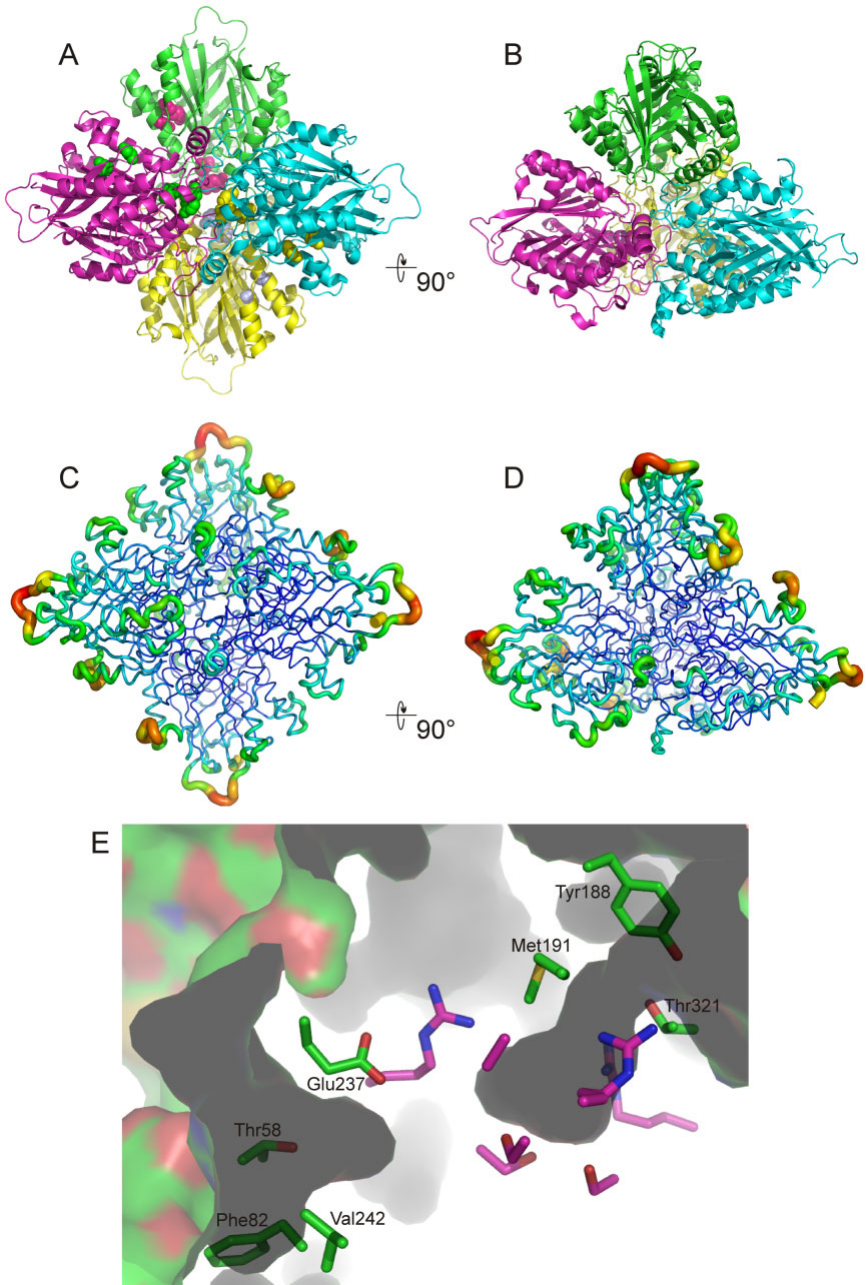
The AtzD tetramer. A and B. Ribbon depictions of the tetrameric structure of AtzD and are approximate 90 degree rotations of each other. In (A) residues 58 and 81 (towards the periphery of the tetramer), and 188 and 191 (towards the centre of the tetramer) are shown as balls in colours that contrast with the main chain; these resides contribute to the two potential substrate channels. C and D. The same rotations, but in this case the chains are coloured by B factors. E. Two channels potentially provide access to the active site of each AtzD monomer. Active-site amino acid residues are shown in pink, but are otherwise unlabelled. The amino acid side-chains that form the channels are shown in green and their identities given.

Gel filtration during purification suggested that the native enzyme was a dimer (data not shown); however, Small Angle X-ray Scattering (SAXS; Supplemental Fig. S2) shows that AtzD forms a compact tetramer with D2 symmetry in solution (Fig. 4A and B). The diameter of the compact tetramer is near identical to that of the dimer, explaining the underestimate in native molecular weight using a lower resolution method (gel filtration) during purification. The core of the D2 tetramer is highly ordered, as indicated by the low B-factors found in the crystal structure (Fig. 4C and D). The surface of the tetrameric protein assembly has two cavities per monomer that could provide access to each active site for substrate ingress/product egress (Fig. 4E). One is formed by residues Tyr188, Met191, Thr321 and Gly345. The second is formed by residues Thr58, Phe82, Glu237 and Val242, and between the two cavities sits Lys162 (hydrogen bonded to the Met84 carbonyl oxygen).

### Active site of AtzD

A phosphate ion was found in a polar, positively charged cavity in the AtzD monomer, making several hydrogen bonds with the protein. This cavity contains three lysine/serine dyads (Lys42, Ser85, Lys162, Ser233, Lys296 and Ser344) any, or all, of which could plausibly form the catalytic site (Botos and Wlodawer, 2007; Ekici *et al*., 2008).

**Fig. 5:**
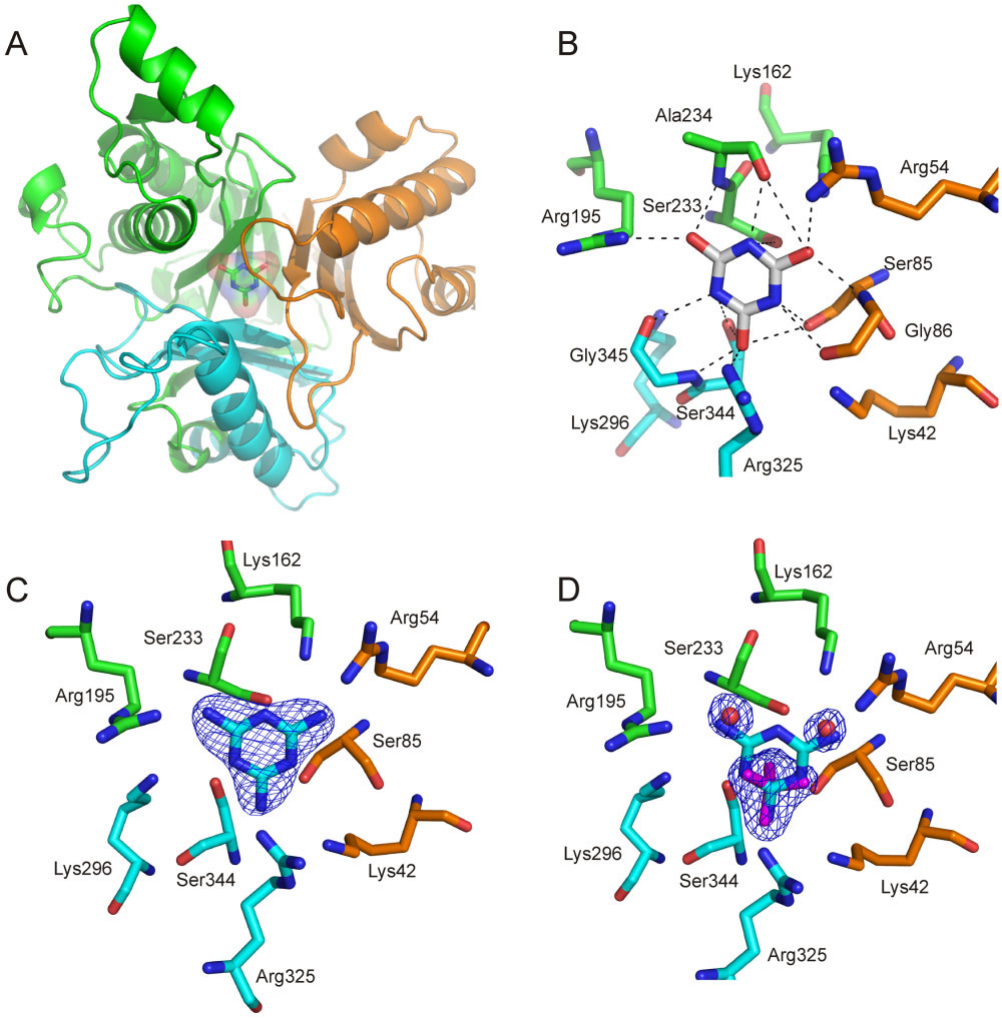
The active site of AtzD. A. The cyanuric acid-bound monomer showing the Toblerone fold (cartoon with RUs coloured as in Fig. 2A) with the substrate bound in the active site (stick and surface). B. Active site of AtzD with cyanuric acid bound. Hydrogen bonds are shown (dashed lines). C. The AtzD active site with bound melamine. D. Electron density for the inhibitor is shown. Density for the phosphate (magenta) and two water molecules (red balls) in the ‘native’ data set with the melamine bound AtzD structure superposed.

Structures of AtzD were then obtained bound to CA or to melamine (1,3,5-triazine-2,4,6-triamine; Table 1, Fig. 5A–C). There was clear density for melamine and CA, displacing two well-ordered water molecules and the phosphate found in the ‘native’ structure (Fig. 5D). Melamine and CA bound in an identical fashion (0.47 Å rmsd) via an extensive hydrogen bonding network between CA/melamine and Arg54, Arg195, Arg325, Gly86, Ala234, Gly345, Ser85, Ser233 and Ser344 (Fig. 5B). BA-containing crystals were also obtained, but at lower resolution. The position of BA superposed well with the position of the melamine and CA.

**Table 1:** Crystallographic statistics

Data collection						
**PDB code**	**3ZGR**	**3ZGT**	**3ZGU**	**3ZGS**		
Ligand	Native	Melamine	Barbituric acid	Cyanuric acid	Xe-derivative	
Spacegroup	R32:H	R32:H	R32:H	R32:H	Spacegroup	R32:H
Cell (a = b × c)	130.7 × 236.9	128.4 × 228.4	129.2 × 233.8	129.1 × 229.7	Cell (a = b × c)	130.7 × 233.1
Resolution (Å)	1.90	2.60	3.09	2.58	Resolution (Å)	2.55
Completeness (%)	99.8 (99.1)	99.9 (99.9)	99.5 (96.5)	100 (100)	Completeness (%)	99.8 (100)
Rmerge %	0.056 (0.573)	0.137 (0.784)	0.145 (0.621)	0.141 (0.799)	Rmerge %	0.101 (0.739)
Mean I/sigI	27.9 (4.4)	12.8 (3.3)	12.9 (3.6)	12.5 (3.1)	Mean I/sigI	21.9 (3.7)
# unique reflections	61 745	22 546	13 929	23 460	# unique reflections	25 199
Multiplicity	11.1	11.1	9.7	11.1	Multiplicity	9.8
					Anomalous completeness	99.8 (100)
					Anomalous multiplicity	5.0
					# Xe	4
**Refinement**					Wavelength (Å)	1.378
Resolution (Å)	102.1–1.90	99.9–2.60	100.9–3.10	100.5–2.58		
No. Reflections	58 623	21 401	13 210	22 272		
Rwork %	17.5 (23.1)	17.9 (25.7)	15.5 (23.6)	18.0 (24.8)		
Rfree %	19.9 (25.1)	21.9 (29.3)	22.3 (26.3)	23.0 (29.0)		
# atoms (total)	5861	5376	5361	5440		
# waters	264	20	5	37		
# metal ions	2	2	2	2		
Mean B value overall (Å^2^)	33.0	53.8	60.0	47.8		
Mean B value inhibitor (Å^2^)	NA	28.2	40.4	37.9		
r.m.s.d. bond lengths (Å^2^)	0.006	0.006	0.006	0.007		
r.m.s.d. bond angles (°)	1.10	1.12	1.10	1.09		
Ramachandran analysis (%) preferred/allowed/outliers	96.9/2.5/0.6	95.4/3.3/1.3	95.3/3.6/1.1	95.1/3.9/1.0		

Values in parentheses are for the high-resolution bin.

**Fig. 6:**
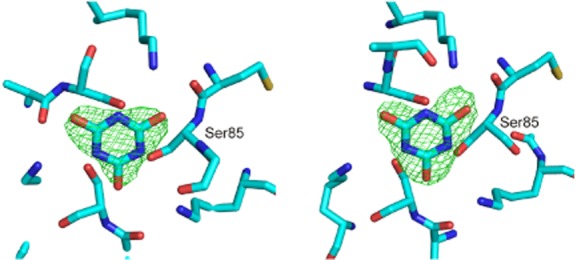
Electron density of cyanuric acid bound in the AtzD active site. Left structure obtained at pH 4.5. Right structure obtained at pH 6.6. In each case Ser85 has been labelled and the electron density obtained for the substrate is shown. In the crystal obtained at pH 4.5 (too low for hydrolysis) the density has a near symmetrical distribution. In the crystal obtained at pH 6.5 (i.e. with hydrolytically active enzyme) the density has become polarized in the vicinity of Ser85, suggesting that Ser85 is the catalytic serine.

The threefold symmetry of the Toblerone fold is also present in the active site (Fig. 5B and C), with each RU contributing an arginine, a serine, a lysine and a main-chain carbonyl (glycine/alanine) to the active site (Fig. 5B and C). Although all three serine/lysine pairs are found in the vicinity of the substrate, only the side-chain of Ser85 is within strong hydrogen bonding distance of the molecules (i.e. < 3 Å). In addition, we were able to detect significant polarization of the substrate's electron density that suggested that a covalent bond could form between the substrate and Ser85 (Fig. 6).

### Identification of catalytic amino acids

Consistent with earlier observations, the optimal pH for the reaction was 8.5–9.0 (Supplemental Fig. S3; Fruchey *et al*., 2003) and similar to other Lys–Ser containing hydrolases, such as the leader peptidase of *E. coli*, which uses a buried lysine with an apparent pK_a_ of 8.7 as a general base to activate a nucleophilic serine (Paetzel *et al*., 1998). Inhibitor studies were also consistent with a Lys–Ser-dependent hydrolytic mechanism, as AtzD was inhibited by phenylmethanesulphonylfluoride (PMSF) and lysine methylation (Table 2).

**Table 2:** Biochemical analysis of AtzD

Inhibitor/treatment	Residual activity (%)
PMSF	8.5
*o*-Phenanthroline	1.2
EDTA	100
ApoAtzD plus Mg^2+^ (50 μM)^a^	60
Lysine alkylation	7.5

a ApoAtzD treated by the addition of 50 μM Zn^2+^, Na^+^, K^+^, Mn^2+^, Co^2+^, Cu^2+^, Ca^2+^ or Fe^2+^ failed to recover any catalytic activity.

Tryptic fingerprinting of AtzD that had been treated with PMSF was used in an attempt to determine which of the active-site serine residues participates in catalysis. In the untreated control, the tryptic fragments of AtzD containing all three active-site serine residues were observed (Supplemental Table S1). When the enzyme was treated with PMSF, the abundance of the Ser85-containing fragment was far lower than in the untreated control, while the other tryptic fragments (including those containing Ser233 and Ser344) do not vary substantively from the control (Supplemental Table S1). Although it was not possible to detect masses equivalent to those expected from the covalently modified Ser85-containing fragments, these observations imply that Ser85 may be the active-site serine, consistent with the crystallographic evidence observed (Fig. 6).

Unexpectedly, an unreported substrate inhibition was observed when substrate was supplied in molar excess (Supplemental Fig. S3; Fruchey *et al*., 2003; Seffernick *et al*., 2012). As it was not possible to delineate the effects of inhibition from the overall rate of the reaction, it was not possible to derive accurate steady-state estimates of *k*_cat_ and *K*_M_. Reactions conducted in the presence of biuret were not inhibited (M. Wilding, unpubl. obs.), suggesting that the inhibition is mediated by either the substrate or 1-carboxybiuret.

AtzD activity was significantly reduced by incubation with *o-*phenanthroline (Table 2), suggesting that the bound metal is required for activity, albeit it appears to be too distant from the substrate-binding pocket to be involved directly in catalysis. Removal of the metal also resulted in a destabilization of the protein (> 80% precipitated at room temperature), which hampered our efforts to obtain a crystal of metal-free AtzD or accurately determine the *K*_d_ value for the bound metal. The activity of apoAtzD could be recovered by providing magnesium (MgCl_2_), but not Zn^2+^ as reported by Soong *et al*. for Bar (Soong *et al*., 2002) or other metal ions (Na^+^, K^+^, Mn^2+^, Co^2+^, Cu^2+^, Ca^2+^ and Fe^2+^). Along with the crystallographic and anomalous scattering data, these observations suggest that the native metal for AtzD is magnesium.

The three potential catalytic dyads were investigated by site-directed mutagenesis. Amino acid substitutions at Lys42 (Ala), Ser85 (Ala), Lys162 (Ala, Arg), Ser233 (Ala), Lys296 (Ala, Arg) and Ser344 (Ala) yielded soluble, but inactive, protein. The unfolding profiles for the variants, as judged by differential scanning fluorimetry (data not shown), were consistent with correctly folded protein. While this provides no direct evidence for the identities of the catalytic residues of AtzD, it does suggest that these residues are critical for catalysis.

### Determinants of substrate specificity

**Fig. 7:**
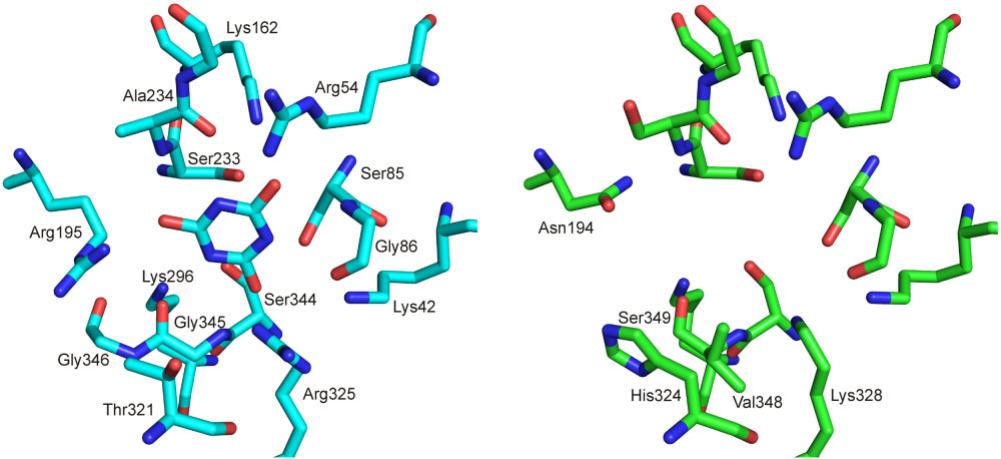
Comparison of the active sites of AtzD and a homology model of Bar. The AtzD active site is shown (left, cyan) with cyanuric acid bound. The modelled Bar active site (right, green) is shown without cyanuric acid docked, only the amino acid residues that differ between AtzD and Bar are indicated on the Bar structure.

AtzD is inhibited by BA and Bar is inhibited by CA, despite the near identical structures of the compounds (Fig. 1A and B). To understand the differences in substrate specificity between AtzD and Bar, the structure of Bar was modelled using the AtzD structure as a template (∼ 44% identical in sequence; Supplemental Fig. S4). The model shows a similar active site with changes in active-site residues being (AtzD to Bar): Arg195/Asn194, Gly345/Val348, Arg325/Lys328, Thr321/His324 and Gly346/Ser349 (Fig. 7). The Gly345Val change is of particular interest as it impinges sterically on the active-site space and introduces a hydrophobic residue in a predominantly hydrophilic pocket.

The major effect of these differences is that the Bar active site does not possess the almost perfect rotational symmetry that AtzD has in the active site, instead Bar has a single axis of bilateral symmetry (Fig. 7). The differences in the active site mirror those of the substrates, in that CA possesses rotational symmetry, while BA does not due to the atomic composition at position 5 in the ring (Fig. 1A and B). The introduction of the hydrophobic surface formed by Val348 in Bar and the loss of positive charge associated with the Arg195-Asn194 substitution in Bar when compared with AtzD also dramatically changes the electronic structure of the active site. It is speculated that these steric and electronic differences underpin the substrate specificity for AtzD and Bar, albeit further data are required to support this hypothesis.

**Fig. 8:**
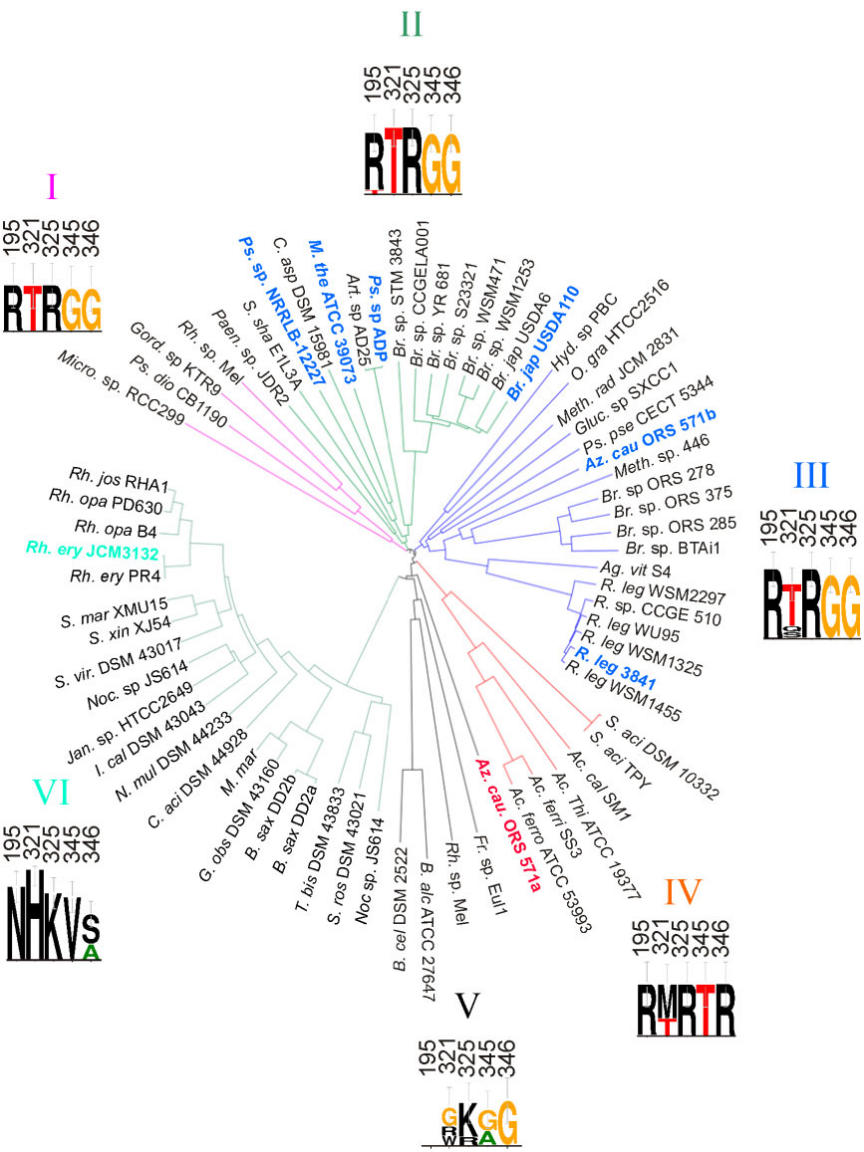
Phylogeny of the AtzD/Bar family. The sequences for the AtzD/Bar homologues in the six groups were sourced from the following organisms (listed in order, clockwise): Group I (purple); *Micromonas* sp. RC299, *Pseudomonas dioxanivorans* CB1190, *Gordonia* sp. KTR8, *Rhodococcus* sp. Mel. Group II (green); *Paenibacillus* sp. JDR2, *Salinisphaera shabanensis* E1L3A, *Pseudomonas* sp. NRRLB-12227, *Clostridium asparagiforme* DSM 15981, *Moorellia thermoacetica* ATCC 39073, *Arthrobacter* sp. AD25, *Pseudomonas* sp. ADP, *Bradyrhizobium* sp. STM3843, *Bradyrhizobium* sp. CCGELA001, *Bradyrhizobium* sp. YR681, *Bradyrhizobium* sp. S23321, *Bradyrhizobium* sp. WSM471, *Bradyrhizobium* sp. WSM1253 *Bradyrhizobium japonicum* USDA6, *Bradyrhizobium japonicum* USDA110. Group III (blue); *Hydrogenophaga* sp. PBC, *Oceanicoia granulosus* HTCC2516, *Methylobacterium radiotolerans* JCM 2831, *Pseudomonas pseudoalcaligenes* CECT 5344, *Azorhizobium caulinodans* ORS 571, *Methylobacterium* sp. 446, *Bradyrhizobium* sp. ORS 278, *Bradyrhizobium* sp. ORS 375, *Bradyrhizobium* sp. ORS 285, *Bradyrhizobium* sp. BTAi1, *Agrobacterium vitis* S4, *Rhizobium leguminosarum* bv. *trifolii* WSM2297, *Rhizobium* sp. CCGE 510, *Rhizobium leguminosarum* bv. *trifolii* WU95, *Rhizobium leguminosarum* bv. *trifolii* WSM1325, *Rhizobium leguminosarum* bv. *viciae* 3841, *Rhizobium leguminosarum* bv. *viciae* WSM1455. Group IV (red): *Sulfobacillus acidophilus* DSM 10332, *Sulfobacillus acidophilus* TPY, *Acidithiobacillus caldus* SM1, *Acidithiobacillus thiooxidans* ATCC 19377, *Acidithiobacillus ferrivorans* SS3, *Acidithiobacillus ferrooxidans* ATCC 27647. Group V (black): *Azorhizobium caulinodans* ORS571, *Frankia* sp. Eul1c, *Rhodococcus* sp. Mel, *Bacillus alcalophilus* ATC 27647, *Bacillus cellulolytitcus* DSM 2552. Group VI (cyan): *Nocardioides* sp. JS614, *Streptosporangium roseum* DSM 43021, *Thermobispora bispora* DSM 43833, *Blastococcus saxobsidens* DD2, *Blastococcus saxobsidens* DD2, *Geodermatophilus obscures* DSM 44928, *Modestobacter marinus*, *Catenulispora acidophia* DSM 44928, *Nakamurella multipartita* DSM 43233, *Intrasporangium calvum* DSM 43043, *Janibacter* sp. HTCC2649, *Nocardioides* sp. JS614, *Saccharomonospora viridis* DSM 43017, *Saccharomonospora xinjiangensis* XJ54, *Saccharomonospora marina* XMU15, *Rhodococcus erythropolis* PR4, *Rhodococcus erythropolis* JCM3132*, Rhodococcus opacus* B4, *Rhodococcus opacus* PD630, *Rhodococcus jostii* RHA1. The identities and conservation of amino acids found in each at positions equivalent to AtzD 195, 321, 325, 345 and 346 are shown as logos. The names of bacterial species from which Toblerone fold enzymes have had their catalytic activities characterized are emboldened and coloured to indicate their substrate specificity: blue for cyanuric acid hydrolase, cyan for barbituric acid hydrolase and red for the absence of hydrolytic activity with either cyanuric acid or barbituric acid.

As residues 195, 321, 325, 345 and 346 in AtzD appear to play a role in determining substrate specificity, the identities of the amino acids at equivalent positions in all 68 known AtzD homologues were analysed and compared with a phylogenetic analysis of these sequences (Fig. 8). Six major phylogenetic groups were discovered. Groups I, II and III possess AtzD-like signatures at positions equivalent to 195, 321, 325, 345 and 346 in AtzD (i.e. Arg195, Thr321, Arg325, Gly345 and Gly346). Indeed, seven characterized CA hydrolases (AtzD and homologues from *Pseudomonas* sp. ADP, *Moorella thermoacetica*, *Pseudomonas* sp. NRRLB-12227, *Bradyrhizobium japonicum* USDA 110, *Rhizobium leguminosarum* bv*. viciae* 3841, *Methylobacterium* sp. 4-46 and locus AZC_3892 from *Azorhizobium caulinodans* ORS 571; Karns, 1999; Seffernick *et al*., 2009; 2012) are located within Groups II and Group III (Fig. 8).

Group VI contains the characterized Bar enzyme and there is strong conservation of a Bar-like signature within this group (i.e. Asn195, His321, Lys325, Val345 and Ser/Ala346). Groups IV and V possess signatures unlike either AtzD or Bar; Group IV has an Arg195, Met/Thr321, Arg325, Thr345 and Arg346 motif, while Group V has low conservation at positions 321 (a Gly, Arg or Trp), 325 (Lys/Arg) and 345 (Gly/Ala) and a conserved Gly at position 346. The equivalent of position 195 is absent in Group V. A single enzyme in Group V has been examined for CA and BA hydrolase activity (locus AZC_3203 from *A. caulinodans* ORS 571; Seffernick *et al*., 2012) and no such activity could be detected. The remaining AtzD active-site residues (Lys42, Lys162, Lys296, Arg54, Arg325, Gly86, Ala234, Ser85, Ser233 and Ser344) were conserved throughout the Toblerone fold proteins (not shown), suggesting that they are not involved in substrate specificity.

## Discussion

### Reaction mechanism

Structural, mutagenesis and biochemical studies suggest that AtzD, and the other members of the Toblerone fold family (TrzD and Bar), are lysine-serine hydrolases. The Michaelis complex is formed when CA binds to AtzD via an extensive hydrogen bonding network (Fig. 5B). Binding to this highly positively charged pocket promotes the formation of the triketide, eliminating the aromaticity of the substrate, with the hydrogen bonding interactions provided by Arg54, Arg195 and Arg325 possibly polarizing the substrate, making the carbonyl carbons more electrophilic and the lactones more scissile. Hydrolysis and formation of the enzyme:acyl intermediate then occurs via nucleophilic attack by a lysine-activated serine (evidenced by inhibitor and pH studies).

A number of potential catalytic mechanisms involving serine and lysine residues are possible, from a simple Ser–Lys dyad to more complex catalytic mechanisms involving multiple serine and lysine residues, with such configurations as Ser–*cis*Ser–Lys, Ser–Xxx–Xxx–Lys and Lys–Ser–Ser–Lys (McKinney and Cravatt, 2003; Ekici *et al*., 2008; Pratt and McLeish, 2010). Although there are three pseudo-equivalent Lys–Ser dyads, it is presumed that a single serine acts as the nucleophile during a single catalytic event, as the product (carboxybiuret; Fig. 1A) is produced via hydrolysis of a single amide bond. It is not possible to definitively attribute the nucleophilic activity to any one of the three active-site serines, and it is possible that any or all three may act as nucleophile (albeit only one can do so *per* catalytic cycle).

**Fig. 9:**
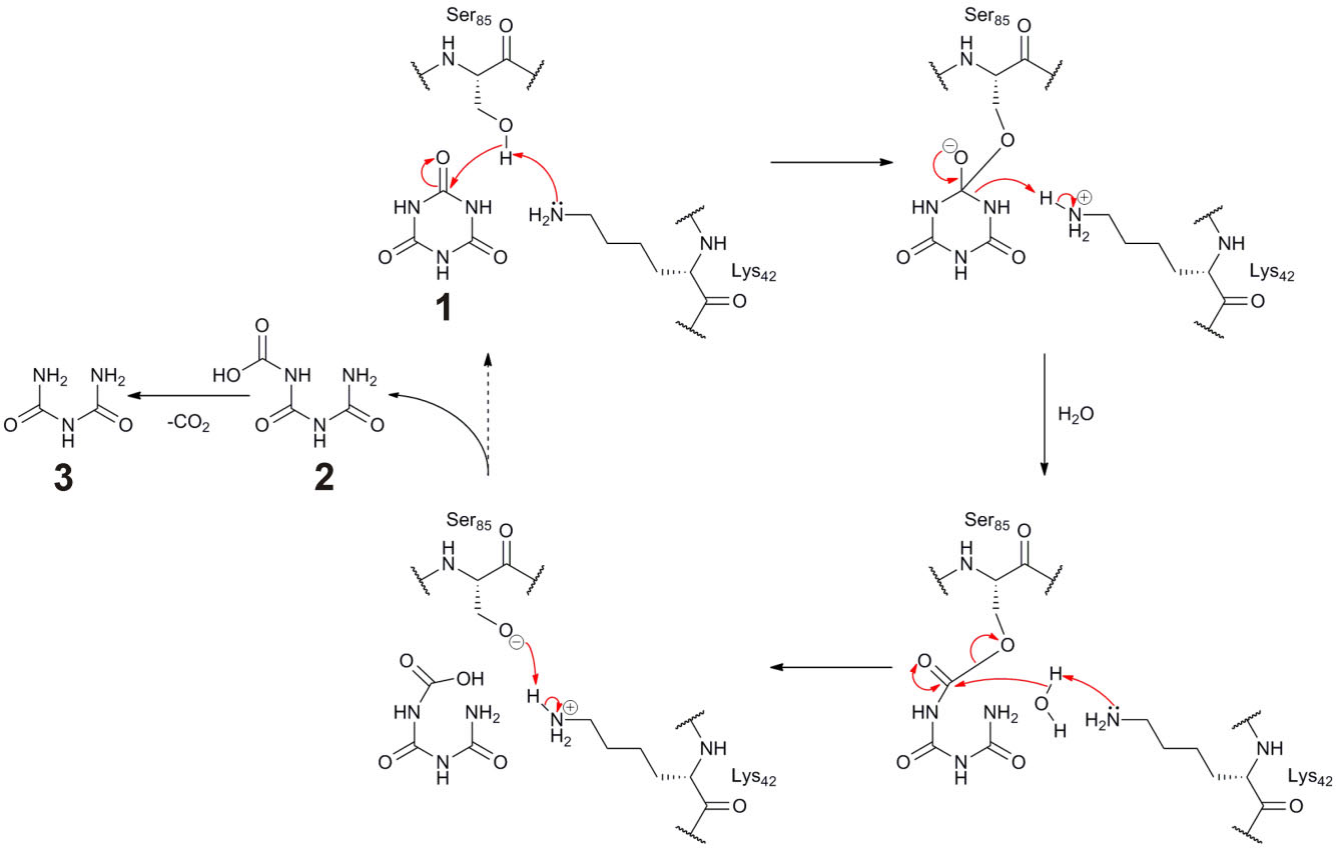
Plausible catalytic mechanism for cyanuric acid hydrolysis by AtzD. Ser85 is activated by Lys42 and acts as nucleophile with bound CA (1), allowing nucleophilic attack at one of the three ketone positions of CA. The tetrahedral intermediate formed undergoes ring opening to form an acyl intermediate. The acylated serine is subsequently hydrolysed by a solvent water molecule, again activated by Lys42, via a tetrahedral intermediate to form carboxybiuret (2) which spontaneously decarboxylates to form biuret (3) and the active site is regenerated by intramolecular proton transfer.

The position of the substrate in the active site (Fig. 5A and B) places Ser85 such that it is closer to a scissile bond than are the other serine residues. Additionally, pH-dependent polarization of the substrate's electron density and a reduction in abundance of the Ser85 containing polypeptide in mass spectra of PMSF-treated tryptic digests of AtzD are suggestive that Ser85 is the dominant (if not sole) nucleophile in the hydrolytic mechanism. A hypothetical reaction mechanism consistent with a Ser85 nucleophile is therefore proposed: Lys42 is a general base, activating Ser85 and promoting formation of a tetrahedral intermediate between Ser85 and the closest substrate carbonyl carbon, this then resolves into the acyl:enzyme intermediate following ring opening (Fig. 9). Thereafter, a solvent water molecule is required to hydrolyse the acyl intermediate and regenerate the serine, liberating carboxybiuret (which then spontaneously decarboxylates to form biuret; Seffernick *et al*., 2012).

### Evolution of the Toblerone fold

To the authors' knowledge AtzD possesses a unique fold, related to, but distinct from, the YgjF-family of trimeric proteins. At some point in their evolutionary history, the subunits of the trimeric ancestor of AtzD and Bar concatenated, possibly following successive gene duplications. On the other hand, the RUs may have been ‘recruited’ from other members of the YgjF protein family. An alternative hypothesis is that an ancestral Toblerone fold protein was truncated to one-third its original length (i.e. a single RU) that was capable of forming stable trimers. However, YgjF-family members are found in all domains of life, catalysing a wide range of reactions and participating in diverse physiological processes (Kim *et al*., 2001; Schaap *et al*., 2002; Burman *et al*., 2007). Until the introduction of *s-*triazine herbicides, the AtzD/Bar enzymes were only known to participate in a single physiological process (BA hydrolysis) in a limited number of species (Seffernick *et al*., 2012). The concatenation scenario is therefore the more likely of the two possible evolutionary histories.

The AtzD active site is located in the central ‘barrel’ of the protein (Fig. 5). In contrast, the three binding pockets of each PSP/RutC trimer are formed by the interactions between adjoining monomers and are located on the outside of the protein (Zhang *et al*., 2010; Knapik *et al*., 2012). In both PSP and RutC, the central barrel provides the core interactions that maintain the trimer (Zhang *et al*., 2010; Knapik *et al*., 2012). In the AtzD family, the strength of these core interactions may have been reduced in an evolutionary trade-off with the optimization of the catalytic function, which could have resulted in a destabilization of the protein. It is plausible that concatenation of the three RUs of AtzD compensates for the loss of the stabilizing core interactions, although it is unclear if concatenation was a prerequisite or a consequence of this trade off.

Removal of the metal ion from AtzD greatly reduced its stability, resulting in protein precipitation at room temperature, suggesting that formation of the metal binding site is also an evolutionary innovation that provides enhanced stability. It is notable that none of the other cations tested appear to substitute for Mg^2+^. Bar has been reported to be Zn^2+^-dependent (Soong *et al*., 2002), despite the fact that the metal binding sites of AtzD and Bar are completely conserved (as it is in 90% of the known AtzD/Bar homologues). Moreover, the metal binding site does not appear well adapted for binding Zn^2+^, albeit the structure of apoAtzD was not determined due to its inherent instability, and it is conceivable that the structure of the metal-binding pockets in holoAtzD/Bar are, in part, determined by the identity of the bound cation. It is also possible that there is a second, unidentified, Zn^2+^ binding site in Bar. However, there are no canonical Zn^2+^-binding motifs in the primary sequence of Bar (Seffernick *et al*., 2012), and so such a site would necessarily be another novel metal binding site.

### Distribution of CA and BA hydrolase activities

It appears that there are six phylogenetically distinct groups within the AtzD/Bar family. The six groups are also separated by the identities of the amino acids that are likely to distinguish substrate specificities of AtzD and Bar. Phylogenetic Group II contains AtzD and TrzD and the predicted determinants of substrate specificity for CA are highly conserved throughout this group (Fig. 8), as they are in Groups I and III. This may suggest that Groups I–III contain CA hydrolases. Group VI contains Bar and the Bar-like specificity signature is highly conserved within this group, suggesting that Group VI contains BA hydrolases. The high level of conservation for the ‘specificity signature’ residues within these groups is consistent with their inferred roles in determining substrate specificity. Moreover, a number of enzymes in Groups II, III and VI have been characterized elsewhere and found to possess activities consistent with the proposed CA or BA hydrolases (Fig. 8; Seffernick *et al*., 2012).

Groups IV and V are unlike the CA hydrolases, BA hydrolases or each other, and may fulfil different physiological roles. Indeed, a single Group V enzyme from *A. caulinodans* ORS571 has been characterized (Seffernick *et al*., 2012), and found to possess neither AtzD-like nor Bar-like substrate specificities. Group V is composed of proteins from five bacterial species (from the genera *Rhizobium*, *Azorhizobium*, *Frankia* and *Bacillus*). There is far lower conservation in the amino acids identified in this study as specificity determinants, suggesting that these enzymes may not share substrate specificities.

It is interesting to note that the Group IV enzymes belong to the genera *Acidothiobacillus* (Gram-negative, γ-proteobacter) and *Sulfobacillus* (Gram-positive, Clostridiales). Although phylogenetically distinct, *Acidothiobacillus* and *Sulfobacillus* are physiologically similar (Waksman and Joffe, 1922; Temple and Colmer, 1951; Golovacheva and Karavaiko, 1978; Kovalenko and Malakhova, 1983; Karavajko *et al*., 1990; Narayan and Sahana, 2009); both are acidophiles that oxidize iron and elemental sulphur, indeed both are exploited in biomining/bioleaching applications (Narayan and Sahana, 2009; Tang *et al*., 2009). It may be that the Toblerone fold enzymes of these two genera fulfil a physiological role that is unique to their unusual physiologies.

### Conclusion

AtzD is the archetype for the Toblerone fold, which has likely evolved by the concatenation of three genes encoding proteins of the YgjF superfamily. The concatenation, along with the evolution of a stabilizing metal binding site (that may also help organize the active site), has allowed the repurposing of the stabilizing central barrel of the monomer for catalysis, which has resulted in a unique family of ring-opening hydrolases. Active-site residues have been identified that differ between AtzD and Bar, and are therefore implicated in determining substrate specificity. The inferred ‘specificity signature’ varies between the six phylogenetic groups of the Toblerone fold family, and is well conserved within Groups I–IV and VI, suggesting that these residues may indeed confer substrate specificities to this family of enzymes.

## Experimental procedures

### DNA manipulation

The *atzD* gene (Accession No. U66917) was provided by GenScript, as an NdeI/BamHI insert in pUC57. The *atzD* gene was subcloned into the NdeI and BamHI sites of pETCC2. The pETCC2 expression vector was a gift from Dr Christopher Coppin (CSIRO Ecosystem Sciences) and is derived from pET14b (Novagen). An in-frame N-terminal hexahis-tag along with thrombin cleavage site (MGSSHHHHHHSSGLVPRGSH) was added to the encoded enzyme as a result of the subcloning.

Mutagenesis was by the over-lapping PCR method of Ho *et al*. (1989) The oligonucleotide primers used are detailed in Supplemental Table S2. Amplicons were resolved on a 0.8% agarose gel and purified using NucleoSpin® Gel kits (Machery Nagel). Purified BamHI and NdeI digested amplicons were cloned into the pETCC2 vector using T4 DNA ligase (NEB). Sequence verification was done by Macrogen (Korea).

### Protein expression and purification

Electrocompetent *E. coli* BL21 λ(DE3) (Invitrogen) were transformed with appropriate plasmids (Suppl Table 2), then grown in Lennox medium (LB; Lennox, 1955) or on LB agar (15 mg ml^−1^ agar, Merck) supplemented with 100 μg ml^−1^ ampicillin (Sigma Aldrich). Starter cultures (50 ml) were grown from single colonies at 37°C for 18 h while being shaken at 200 r.p.m., used to inoculate 950 ml of LB and incubated at 37°C to an OD_600_ of 0.6–0.8. Cultures were induced by addition of 100 μM isopropyl-beta-d-thiogalactopyranoside (IPTG; Astral) and incubated at 37°C overnight while shaking at 200 r.p.m. Cultures were then harvested by centrifugation (4000 *g*, 10 min) in an Avanti J-E centrifuge (Beckman Coulter) and resuspended in lysis buffer (50 mM sodium phosphate pH 7.5, 50 mM NaCl) and lysed by passing through Avestin C3 homogenizer three times at 18 000 p.s.i. Insoluble material was removed by centrifugation (18 000 *g*) using an Avanti J-E centrifuge.

Metal ion affinity chromatography (Ni-NTA Superflow Cartridge; Qiagen) was used as per the manufacturer's instructions. AtzD eluted at 160 mM imidazole. AtzD was further purified by size exclusion chromatography using a 130 ml column packed with Superdex 200 prep grade resin (GE Healthcare Life Sciences) using lysis buffer. AtzD was estimated to be > 98% pure by Coomassie (Sigma Aldrich) stained precast SDS-PAGE gel (4–20% Tris-HEPES-SDS, Thermo Scientific), and typical yields were 7.5–8.5 mg from 1 l of culture.

### Crystallization

Crystals of native AtzD were prepared as follows: concentrated protein (10.4 mg ml^−1^ in lysis buffer) was set up in crystallization droplets consisting of 200 nl protein solution, 250 nl crystallization cocktail and 50 nl additive solution [0.1 M sodium HEPES pH 6.9, 0.2 M NH_4_Cl, 20% (w/v) polyethylene glycol (PEG) 6000]. The crystallization cocktail contained a low-molecular-weight PEG (either PEG 300, PEG monomethyl ether 350 or PEG 400) between 35% and 50% (v/v) in sodium phosphate (50–100 mM; pH 6.5–8) buffer, with or without NaCl (100–200 mM). All chemicals were obtained from Sigma Chemicals (St. Louis, USA). Droplets were set up with a Phoenix nanodispensing robot (ARI, Sunnyvale, CA) into SD-2 plates (Molecular Dimensions, UK) and were stored at 20°C. Triangular prism-shaped crystals appeared within 24 h, and grew large enough to obtain X-ray data within a week (Supplemental Fig. S5).

Substrate/inhibitor bound AtzD crystals were prepared as follows: AtzD protein in 50 mM sodium phosphate pH 7.5, 50 mM NaCl was dialysed against HEPES buffer (50 mM, pH 7.5) containing NaCl (50 mM), DTT (5 mM) and melamine (10 mM), barbituric acid (10 mM) or cyanuric acid (0.1 mM) and then set up in crystallization experiments as described above, against crystallization conditions without phosphate.

### Crystal data collection

Crystals were taken to the MX-2 beamline of the Australian Synchrotron and cryo-cooled to 100 K directly in the nitrogen stream. A xenon derivative was prepared by exposing a crystal to xenon gas at 20 p.s.i. for 2 min, and flash-cooled in liquid nitrogen. A total of 720 frames of 0.5° oscillation were used to collect 360 degrees of data for the Xe derivative data set. All further data sets consisted of 360 frames of 0.5° oscillation for a total of 180 degrees of data. Each was processed as described below. Melamine, barbituic acid and cyanuric acid were all placed in clear density using Afitt (OpenEye Scientific Software, USA) for those data sets collected after co-crystallization with these compounds.

Anomalous data were collected at 1.7149 Å (7230 eV) on 17 isomorphous AtzD native crystals and the best data were merged to give a data set with 140-fold multiplicity which gave exquisite anomalous difference maps, showing every sulphur atom, the phosphate ions as well as the two metal binding sites (Supplemental Fig. S1).

### Structural determination

Data sets were indexed with XDS (Kabsch, 2010) and processed with SCALA (Evans, 2006), and entered into the SIRAS protocol in Auto-Rickshaw (Panjikar *et al*., 2005) to locate the four xenon atoms. Phases from this derivative were sufficient to allow Buccaneer (Cowtan, 2006) to autobuild a partial model, which was used in Phaser (McCoy *et al*., 2007) as a molecular replacement model (crystallographic statistics are found in Table 1). These data allowed the complete tracing of the chain from residue 2 to residue 364, using the program coot (Emsley *et al*., 2010). The model was refined using Refmac (Murshudov *et al*., 1997) to a final resolution of 1.90 Å, and showed clean density for the entire chain excepting weak density for the residues of the loop from residues 105 to 109. This model was subsequently used for molecular replacement using Phaser (McCoy *et al*., 2007) for subsequent data sets with inhibitors bound.

### Small-angle X-ray scattering (SAXS)

AtzD was dialysed overnight into a 50 mM phosphate buffer, 50 mM NaCl, 1 mM DTT (pH 7.5). The same buffer was used as the buffer standard during data collection. A dilution series of AtzD (from 0.19 to 3 mg ml^−1^) was prepared, and scattering data were collected for 1 s using a Pilatus 1 M photon counting detector (Dektris) with a sample to detector distance of 1.6 m. Ten replicate images were collected for each sample and averaged, with outlier rejection, to control for radiation damage. Data were measured in a Q range from 0.01 to 0.5 Å^−1^ and at the highest protein concentration the scattering remained above the noise threshold to the edge of the detector.

AtzD (75 μl, 6 mg ml^−1^) was injected on to a size exclusion column (Wyatt Silica Bead column, 300 Å pore size) that had been pre-equilibrated with the PO_4_/NaCl buffer. The column was developed at 0.2 ml min^−1^, and a single peak eluted. The SAXS scattering showed no change over the peak (data not shown). A CRYSOL (Svergun *et al*., 1995) fit of various quaternary structures for the protein to the scattering data was performed (Supplemental Fig. S2).

### Kinetic analysis

Hydrolysis of CA and BA was assayed by UV-visible spectroscopy as described elsewhere (Seffernick *et al*., 2012), using a SpectraMax M2 spectrophotometer (Molecular Devices). Reactions [200 μl in 1 mM TAPS (N-Tris(hydroxymethyl)methyl-3-aminopropanesulphonic acid; pH 8.5); 1 μM AtzD] with 10–1000 μM substrate were assayed in UV-transparent 96-well microtitre plates (Greiner Bio-one). Absorbances were measured at 15 s intervals for 10 min. The pH-dependence of AtzD was determined in MOPS [3-(N-morpholino)propanesulphonic acid; 1 mM, pH 6.5, 6.9 and 7.5], TAPS (1 mM pH 7.5, 7.9 and 8.5) and CHES (*N*-Cyclohexyl-2-aminoethanesulphonic acid; 1 mM pH 9).

### Enzyme inhibition

Alkylation of lysines in AtzD was carried out using JBS Methylation Kit (Jena Bioscience) following manufacturer's guidelines. A protein concentration of 6 mg ml^−1^ in sodium phosphate (50 mM, pH 7.5)/NaCl buffer (50 mM) was used at the time of treatment. Phenylmethylsulphonyl fluoride (PMSF) was prepared at 100 mM in isopropanol. Enzyme solutions were prepared at 4 μM concentrations in MOPS buffer (1 mM, pH 6.5) and treated twice with PMSF (1 mM final concentration) for 30 min. Trypsin was used to as a positive control using *N*_α_-Benzoyl-l-arginine ethyl ester hydrochloride (BAEE) as a control substrate. Trypsin activity was monitored by UV spectrophotometrically at 253 nm.

Ethylenediaminetetraacetic acid (EDTA; 20 mM) and *o-*phenanthroline (75 mM) in 25 mM MOPS buffer (pH 8.5) were used to dialyse AtzD (117 μM) overnight at 4°C. Chelators were removed by centrifugation in a 30 kDa spin column (Amicon Ultra, Millipore). The zinc amidohydrolase TrzN (E.C. 3.8.1.8) was used as a positive control. Metals (Mg^2+^ Na^+^, K^+^, Mn^2+^, Co^2+^, Cu^2+^, Ca^2^, Zn^2+^ and Fe^2+^; 50 μM) were added to *o*-phenanthroline treated ApoAtzD and incubated at 4°C for 16 h.

### Mass spectrometry

Peptides generated from tryptic digestions of AtzD were acidified with 0.5% formic acid, filtered [Millex-LG, low protein binding hydrophilic LCR (PTFE), 0.2 μm], and analysed by nanoflow, reversed phase, liquid chromatography-tandem mass spectrometry (MS) using an Agilent Chip Cube system coupled to an Agilent XCD ion trap mass spectrometer. MSMS spectra were assigned to sequences by several rounds searching and ‘autovalidation’ using SpectrumMill software (Agilent Rev A.03.03.084 SR4). First a small database of common contaminant sequences such as trypsin and keratins was searched assuming tryptic cleavages. Remaining unassigned spectra were used to search just the AtzD sequence allowing trypsin- and chymotrypsin-specific cleavages. The trypsin (Sigma-Aldrich) had not been treated to prevent chymotrypsin-specific cleavages and this generated some mixed tryptic/chymotryptic cleavages that usefully extended the sequence coverage. Finally, remaining unassigned spectra were used to search the AtzD sequence in ‘homology’ mode allowing single unassigned mass gaps in sequences that were otherwise consistent with MS/MS spectra. Spectra assigned in this last mode were inspected individually to verify putative modifications of amino acid residues.

### Modelling of Bar

Bar was modelled using the Swiss-Model software as found on the web server (Arnold *et al*., 2006); the AtzD structure was used as the starting point (44% sequence identity over 364 residues). There are some minor differences between the structures in various loops: residues 74–78 (AtzD) has an extra residue in the AtzD loop; residues 106–111 (AtzD) this loop of weak density in the AtzD structure is built slightly differently in the Bar structure; residues 210–215 (AtzD) has two extra residues in the AtzD loop; residues 280–283 (AtzD) has six extra residues in the Bar modelled loop; the AtzD structure has a single amino acid residue extension at the C-terminus of the protein as well.

### *In silico* sequence analysis

AtzD homologues were identified via a BlastP search of non-redundant databases. The following sequences were returned with *E*-values of less than 10^−30^ and identity scores of greater than 40%, there were no other hits with *E*-values of less than 10^1^: NP862537 (*Pseudomonas* sp. ADP), ABK41866 (*Arthrobacter* sp. AD25), YP430955 (*M. thermoacetica* ATCC 39073), ZP10581004 (*Bradyrhizobium* sp. YR681), ZP09650932 (*Bradyrhizobium* sp. WSM471), P0A3V4 (*Pseudomonas* sp. NRRLB-12227), ZP10083023 (*Bradyrhizobium* sp. WSM1253), YP005453208 (*Bradyrhizobium* sp. S23321), YP005606973 (*B. japonicum* USDA 6), ZP09433530 (*Bradyrhizobium* sp. STM 3843), EJZ29306 (*Bradyrhizobium* sp. CCGE-LA001), NP773921 (*B. japonicum* USDA 110), ZP03758143 (*Clostridium asparagiforme* DSM 15981), YP003013624 (*Paenibacillus* sp. JDR-2), YP001757420 (*Methylobacterium radiotolerans* JCM 2831), ZP10760424 (*Pseudomonas pseudoalcaligenes* CECT 5344), ZP08551100 (*Salinisphaera shabanensis* E1L3A), ZP08318418 (*Gluconacetobacter* sp. SXCC-1), YP004783181 (*Acidithiobacillus ferrivorans* SS3), YP002219377 (*Acidithiobacillus ferrooxidans* ATCC 53993), YP001526119 (*A. caulinodans* ORS 571), YP001208170 (*Bradyrhizobium* sp. ORS 278), YP002547456 (*Agrobacterium vitis* S4), YP004750255 (*Acidithiobacillus caldus* SM-1), EIW44662 (*R. leguminosarum* bv*. trifolii* WU95), YP770629 (*R. leguminosarum* bv*. viciae* 3841), EJC71551 (*R. leguminosarum* bv*. viciae* WSM1455), YP002979447 (*R. leguminosarum* bv*. trifolii* WSM1325), ZP09420969 (*Bradyrhizobium* sp. ORS 375), YP001526808 (*A. caulinodans* ORS 571), ZP10837886 (*Rhizobium* sp. CCGE 510), EJC83804 (*R. leguminosarum* bv*. trifolii* WSM2297), ZP09473782 (*Bradyrhizobium* sp. ORS 285), YP001237458 (*Bradyrhizobium* sp. BTAi1), ZP01155857 (*Oceanicola granulosus* HTCC2516), ZP09997547 (*Acidithiobacillus thiooxidans* ATCC 19377), ZP10152173 (*Hydrogenophaga* sp. PBC), YP006671162 (*Gordonia* sp. KTR9), YP005257446 (*Sulfobacillus acidophilus* DSM 10332), YP004719285 (*S. acidophilus* TPY), YP001770627 (*Methylobacterium* sp. 4-46), AEX65082 (*Rhodococcus* sp. Mel), YP00433323 (*Pseudonocardia dioxanivorans* CB1190), XP002503480 (*Micromonas* sp. RCC299), CAC86669 (*Rhodococcus erythropolis*), YP004017027 (*Frankia* sp. EuI1c), YP004094229 (*Bacillus cellulosilyticus* DSM 2522), YP003112640 (*Catenulispora acidiphila* DSM 44928), YP002769329 (*R. erythropolis* PR4), YP922706 (*Nocardioides* sp. JS614), YP002779991 (*Rhodococcus opacus* B4), YP005331388 (*Blastococcus saxobsidens* DD2), ZP00996765 (*Janibacter* sp. HTCC2649), YP003133937 (*Saccharomonospora viridis* DSM 43017), YP005327200 (*B. saxobsidens* DD2), YP006364153 (*Modestobacter marinus*), ZP09986349 (*Saccharomonospora xinjiangensis* XJ-54), YP003204583 (*Nakamurella multipartita* DSM 44233), ZP09740740 (*Saccharomonospora marina* XMU15), EHI43897 (*R. opacus* PD630), YP004098318 (*Intrasporangium calvum* DSM 43043), YP003407358 (*Geodermatophilus obscurus* DSM 43160), YP003341997 (*Streptosporangium roseum* DSM 43021), YP705235 (*Rhodococcus jostii* RHA1), YP925454 (*Nocardioides* sp. JS614), YP003652816 (*Thermobispora bispora* DSM 43833), ZP10819722 (*Bacillus alcalophilus* ATCC 27647), AEX65050 (*Rhodococcus* sp. Mel).

Alignments and bootstrapping (1000×) of the AtzD superfamily sequences were carried out using clustalw (http://www.bioinformatics.nl/tools/clustalw.html). A phylogenetic tree was constructed from this alignment using the Interactive Tree of Life (iTol; Letunic and Bork, 2007;2011) and consensus sequences were created using Weblogo sequence generator (Crooks *et al*., 2004).

### Accession codes

The molecular models for AtzD and AtzD bound to CA, melamine and BA were deposited in the Protein Data Bank, with PDB codes 3ZGR, 3ZGS, 3ZGT and 3ZGT.
