# Supplementary material for: Cyanuric acid hydrolase: evolutionary innovation by structural concatenation
Source: Mol Microbiol. 2013 May 20;88(6):1149–63. doi: 10.1111/mmi.12249 (PMC3758960; doi:10.1111/mmi.12249)

**Differential Scanning Fluorimetry of AtzD and its active site variants**

The data from DSF experiments are provided here for the reviewers. In each experiment the variants were analysed with 8-fold replication, with the wild-type as control in each case.

The first derivatives are shown, and there is a clear difference in apparent Tm between the wild-type and variants. Unexpectedly, in some of the variants the change in Tm indicates that the amino acid substitution has caused a significant gain in stability. We do not believe that these unusal observations make a material difference to work described in the submitted manuscript: i.e. the proteins appear to be folded. However, we would like to better understand the cause of the unexpected increases in Tm before releasing these data.

**Serine to alanine substitutions:**

Light green – S85A   (49.5-50C)

Teal – S233A   (53.5C)

Green - S344 A (46.5-47C)

Red – WT  (55.0C)


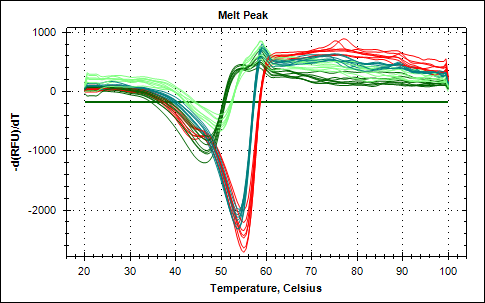


**Lysine to alanine substitutions:**

K42A – light pink  (40C)

K163A – magenta (65.5C)

K296A – oxblood (62-63C)

WT – red  (55C)


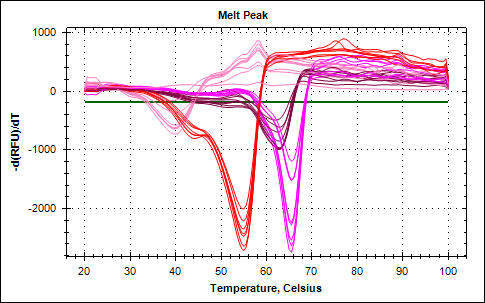


**Lysine to arginine substitutions:**

K163R – cyan (57C)

K296R – blue (38.5-39C)

WT – red  (55C)


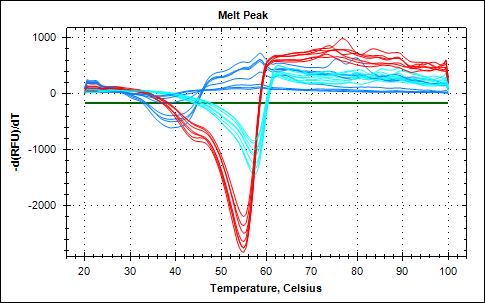

Supplement: Supplementary file 1 [file mmi0088-1149-SD1.zip › mmi_12249_Differential Scanning Fluorimetry of AtzD and its active site variants.docx]
