# Supplementary material for: Cyanuric acid hydrolase: evolutionary innovation by structural concatenation
Source: Mol Microbiol. 2013 May 20;88(6):1149–63. doi: 10.1111/mmi.12249 (PMC3758960; doi:10.1111/mmi.12249)
Supplement: Supplementary file 1 [file mmi0088-1149-SD1.zip › mmi_12249_Suppl_fig_1.docx]

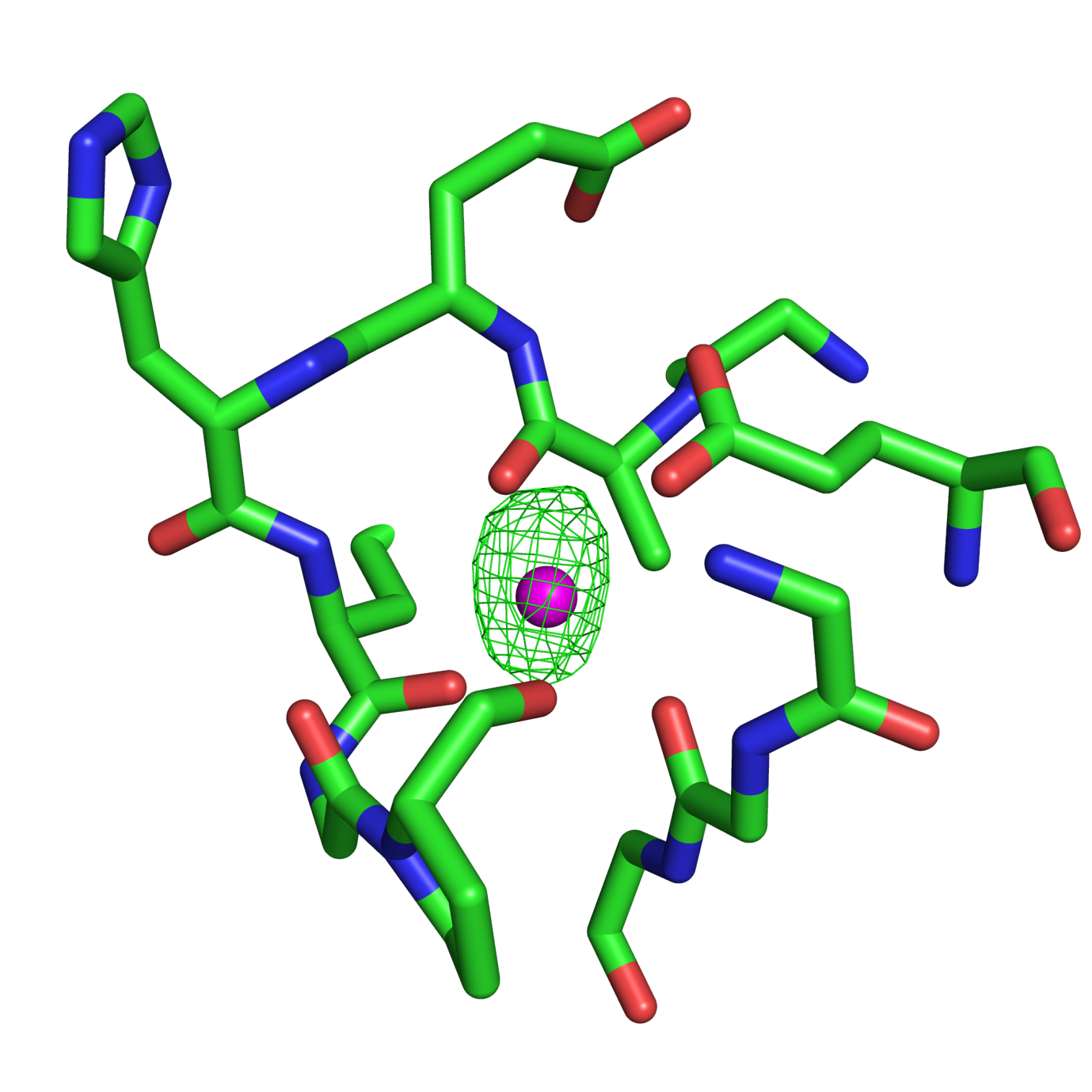

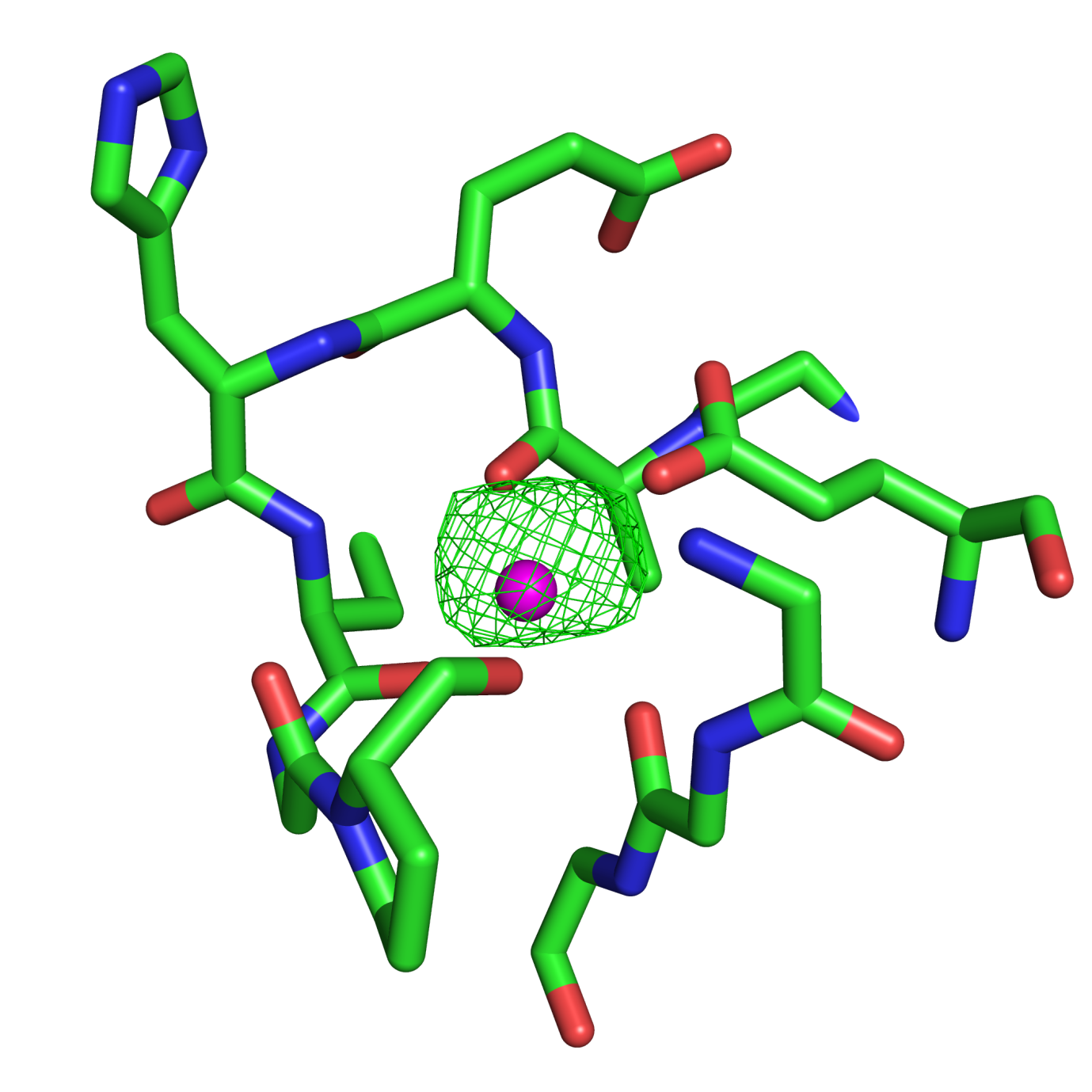


**Supplemental Figure 1. Anomalous scattering for the two bound metals in the asymmetric unit.** Top is the anomalous difference map (green mesh) set at 4.0 sigma at the metal site in protomer B of the asymmetric unit. Bottom is the anomalous difference map at 4.0 sigma at the metal site of protomer A. These sites were initially modelled as magnesium ions, but could also be sodium ions as sodium and magnesium are indistinguishable by this method. The anomalous difference maps were generated from data collected at 1.7149 Å (7230 eV) with 140 fold average redundancy. The orientation of both is approximately the same as that shown in Figure 3B.
