# Supplementary material for: Cyanuric acid hydrolase: evolutionary innovation by structural concatenation
Source: Mol Microbiol. 2013 May 20;88(6):1149–63. doi: 10.1111/mmi.12249 (PMC3758960; doi:10.1111/mmi.12249)
Supplement: Supplementary file 1 [file mmi0088-1149-SD1.zip › mmi_12249_Suppl_fig_2.docx]

**Supplemental Figure 2.** Panels 1-4 show CRYSOL fits of different oligomeric states of the AtzD monomer, plotted against the measured SAXS data. Both the D2 and C4 (back to back) tetramers are found in the crystal, although only a dimer is found in the asymmetric unit.

Panel 5 shows a plot of the total forward scatter vs. the concentration for the SAXS dilution series, showing an extremely good fit to the D2 tetramer with no variation across the concentration range.
