# Supplementary material for: Cyanuric acid hydrolase: evolutionary innovation by structural concatenation
Source: Mol Microbiol. 2013 May 20;88(6):1149–63. doi: 10.1111/mmi.12249 (PMC3758960; doi:10.1111/mmi.12249)
Supplement: Supplementary file 1 [file mmi0088-1149-SD1.zip › mmi_12249_Suppl_Fig_3.docx]

**Supplemental Figure 3.** (Top) pH dependence of AtzD-dependent hydrolysis of cyanuric acid. (Bottom) Substrate inhibition of AtzD-dependent hydrolysis of cyanuric acid at pH 9.0. A relative rate of 1 is equivalent to the rate of AtzD (1µM) at pH8.5 at 50 µM cyanuric acid (30 µM.sec^-1^).
