# Supplementary material for: Cyanuric acid hydrolase: evolutionary innovation by structural concatenation
Source: Mol Microbiol. 2013 May 20;88(6):1149–63. doi: 10.1111/mmi.12249 (PMC3758960; doi:10.1111/mmi.12249)
Supplement: Supplementary file 1 [file mmi0088-1149-SD1.zip › mmi_12249_Suppl_Fig_4_(Revised).docx]

**42**

**|**

**AtzD -MYHIDVFRIPCHSPGDTSGLEDLIETGRVAPADIVAVMGKTEGNGCVND**

**BAR MPEAIEVRKVPLHSVSDASELAKLIDDGVLEADRVIAVIGKTEGNGGVND**

*

**54 85**

**| |**

**AtzD**  **YTREYATAMLAACLGRHLQLPPHEVEKRVAFVMSGGTEGVLSPHHTVFAR**

**BAR**  **YTRIIADRAFREVLSAKGNRSPEEVAE-VPIVWSGGTDGVISPHATIFAT**

* **

**AtzD**  **RPAIDAHRPAGKRLTLGIAFTRDFLPEEIGRHAQITETAGAVKRAMRDAG**

**BAR**  **VPADKVTKTDEPRLTVGVAMSEQLLPEDIGRTAMITKVAAAVKDAMADAG**

**162 195**

**| |**

**AtzD**  **IASIDDLHFVQVKCPLLTPAKIASARSRGCAPVTTDTYESMGYSRGASAL**

**BAR**  **ITDPADVHYVQTKTPLLTIHTIRDAKSRGKTVWTEQTHESMDLSNGGTAL**

* *

!

**233**

**|**

**AtzD**  **GIALATEEVPSSMLVDESVLNDWSLSSSLASASAGIELEHNVVIAIGMSE**

**BAR**  **GIAVALGEID--MPTDEDVMHSRELFSSVASCSSGVELDRAQIVVVGNAR**

**

**AtzD**  **QATSELVIAHGVMSDAIDAASVRRTIESLGIR------SDDEMDRIVNVF**

**BAR**  **GVGGRYRIGHSVMKDPLDQDGIWAAIRDAGLELPERPHSNDLDGQLVNVF**

**296 321 344**

**| | |**

**AtzD**  **AKAEASPDGVVRGMRHTMLSDSDINSTRHARAVTGAAIASVVGHGMVYVS**

**BAR**  **LKCEASQDGTVRGRRNAMLDDSDVHWHRQIKSCVGGVTAAVTGDPAVFVS**

* m !* *

**345 350 355**

**| | |**

**AtzD**  **GGAEHQGPAGGGPFAVIARA--**

**BAR**  **VSAAHQGPEGGGPVAAIVDLGQ**

*!m m m m

!

**Supplemental Figure 4. Alignment of AtzD and Bar.** Where possible residues referred to in the results and discussion are numbered (above). Symbols below the sequence indicate the positions of active site residues (*), resides that appear to determine AtzD and Bar substrate specificities (!) and residues that form the metal binding site (m).
