# Supplementary material for: Cyanuric acid hydrolase: evolutionary innovation by structural concatenation
Source: Mol Microbiol. 2013 May 20;88(6):1149–63. doi: 10.1111/mmi.12249 (PMC3758960; doi:10.1111/mmi.12249)
Supplement: Supplementary file 1 [file mmi0088-1149-SD1.zip › mmi_12249_Suppl_Fig_5.docx]

.
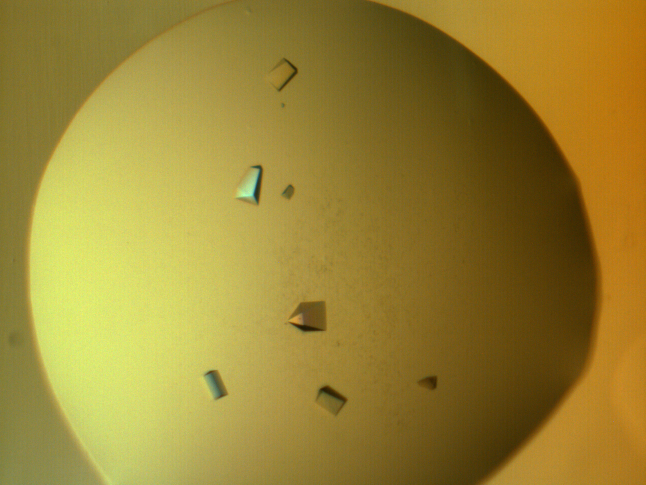


**Supplemental Figure 5. Crystallization of native AtzD.** Crystal of native AtzD in PEG 400. The crystal in the centre of the image is 0.1 mm x 0.1 mm
