# Supplementary material for: Cyanuric acid hydrolase: evolutionary innovation by structural concatenation
Source: Mol Microbiol. 2013 May 20;88(6):1149–63. doi: 10.1111/mmi.12249 (PMC3758960; doi:10.1111/mmi.12249)
Supplement: Supplementary file 1 [file mmi0088-1149-SD1.zip › mmi_12249_Suppl_Table_1.docx]

| Treatment | No. spectra | No. Distinct peptides | % amino acid coverage | Total Protein Spectral Intensity | Intensity of peptide (R77)VAF…TVF(A) |
| --- | --- | --- | --- | --- | --- |
| Control | 55 | 16 | 44 | 7.9 x 10^9^ | 8 x spectra totaling 5.7x10^8^ |
| PMSF | 14 | 10 | 34 | 1.7 x 10^9^ | 1 spectrum at 5.0 x 10^6^ |

**Supplemental Table 1. Tryptic finger printing of AtzD covalently modified with PMSF.** Peptide V78AF…TVF87 includes the putative active site serine residue (Ser85).
