# Supplementary material for: Cyanuric acid hydrolase: evolutionary innovation by structural concatenation
Source: Mol Microbiol. 2013 May 20;88(6):1149–63. doi: 10.1111/mmi.12249 (PMC3758960; doi:10.1111/mmi.12249)
Supplement: Supplementary file 1 [file mmi0088-1149-SD1.zip › mmi_12249_Suppl_Table_2.docx]

**Supplemental Table 2. Plasmids and Primers.** Site-directed mutations shown in red.

| **Plasmid/primer** | **Detail/sequence (5’-3’)** |
| --- | --- |
| **pUC57:*atzD*** | pUC57 containing the *atzD* gene based on Genbank Acc. No. U66917 (GenScript USA Inc.) |
| **pETcc2** | Expression vector based on pET14b (see methods for details) |
| **pETCC2 Rev** | TCATCGTCATCCTCGGCACCGTCA |
| **K42A Fwd** | ATCGTCGCGGTAATGGGCGCTACCGAGGGCAATGGCTG |
| **K42A Rev** | GCAGCCATTGCCCTCGGTAGCGCCCATTACCGCGACGA |
| **K42R Fwd** | CGCGGTAATGGGCAGAACCGAGGGCAATG |
| **K42R Rev** | CCATTGCCCTCGGTTCTGCCCATTACCGC |
| **S85A Fwd** | TCGCGTTTGTGATGGCAGGTGGGACGGAAG |
| **S85A Rev** | TTCCGTCCCACCTGCCATCACAAACGCGAC |
| **K162R Fwd** | CATTTTGTGCAGGTGCGATGTCCGCTGCTGACAC |
| **K162R Rev** | TGTCAGCAGCGGACATCGCACCTGCACAAAATGC |
| **K162A Fwd** | TGCATTTTGTGCAGGTGGCATGTCCGCTGCTGACAC |
| **K162A Rev** | GGTGTCAGCAGCGGACATGCCACCTGCACAAAATGC |
| **S233A Fwd** | ATCGTCACTGGCGTCGGCGGCAGCAGGCATCGAACTGGAG |
| **S233A Rev** | GCTCCAGTTCGATGCCTGCTGCCGCCGACGCCAGTGACGA |
| **S344A Fwd** | GGCATGGTGTATGTGGCAGGTGGCGCCGAGCATC |
| **S344A Rev** | ATGCTCGGCGCCACCTGCCACATACACCATGCCA |
| **K296R Fwd** | AACGTATTCGCCAGAGCGGAGGCGAG |
| **K296R Rev** | GCTCGCCTCCGCTCTGGCGAATACGT |
| **K296A Fwd** | TCAACGTATTCGCCGCAGCGGAGGCGAGC |
| **K296A Rev** | GGCTCGCCTCCGCTGCGGCGAATACGTTG |
